# Do insect food-derived ecdysteroids appear in the blood of waterfowl, predatory birds, carnivorous mammals and primates?

**DOI:** 10.1371/journal.pone.0357290

**Published:** 2026-09-02

**Authors:** Sándor Hornok, Róbert Berkecz, Andor Pitó, Paula Baro, Kathrin M. Röper, József Lanszki, Alexandra Biácsi, Károly Erdélyi, Endre Sós, Viktor Molnár, Endre Papp, Sándor Boldogh, Solt Szabolcs, Gergő Keve, Attila Hunyadi

**Affiliations:** 1 Department of Parasitology and Zoology, University of Veterinary Medicine, Budapest, Hungary; 2 HUN-REN UVMB Climate Change: New Blood-sucking Parasites and Vector-borne Pathogens Research Group, Budapest, Hungary; 3 Institute of Pharmaceutical Analysis, University of Szeged, Szeged, Hungary; 4 Department of Forensic Medicine, Albert Szent-Györgyi Clinical Center, University of Szeged, Szeged, Hungary; 5 Hannover Zoo, Hannover, Germany; 6 HUN-REN Balaton Limnological Research Institute, Tihany, Hungary; 7 Nyíregyházi Állatpark Nonprofit Kft. (Sosto Zoo), Nyíregyháza, Hungary; 8 HUN-REN Veterinary Medical Research Institute, Budapest, Hungary; 9 Department of Microbiology and Infectious Diseases, University of Veterinary Medicine, Budapest, Hungary; 10 Budapest Zoo and Botanical Garden, Budapest, Hungary; 11 Department of Exotic Animal, Wildlife, Fish and Honeybee Medicine, University of Veterinary Medicine, Budapest, Hungary; 12 Aggtelek National Park Directorate, Jósvafő, Hungary; 13 Department of Evolutionary Zoology and Human Biology, University of Debrecen, Debrecen, Hungary; 14 Institute of Pharmacognosy, University of Szeged, Szeged, Hungary; 15 HUN-REN-SZTE Biologically Active Natural Products Research Group, Szeged, Hungary; University of Kotli, PAKISTAN

## Abstract

Arthropod molting hormones, the so-called ecdysteroids were experimentally shown to have anabolic effects in higher vertebrates, promoting energy supply, protein synthesis, and tissue growth. Insectivorous songbirds (Aves: Passeriformes) and bats (Mammalia: Chiroptera) were demonstrated to have naturally acquired ecdysteroids in their blood. However, it is not known if partly insectivorous or artificially insect-fed vertebrates would also have ecdysteroids in their circulation. The aim of this screening study was to clarify this, by collecting blood samples of mammals, birds and reptiles that are wild-living or receive insect diet at zoological gardens, followed by ultra-high performance liquid chromatography and high-resolution quadrupole-orbitrap mass spectrometry analyses. The zoo animal samples originated from one reptilian species (n = 3), as well as one species of insectivorous mammals (n = 8), three species of carnivores (n = 17) and one species of primates (n = 2). Three further species of road-killed mammalian carnivores were sampled *postmortem* (n = 15). Finally, blood samples were also drawn and analyzed from six species of water-associated (n = 44) and three species of predatory birds (n = 47). Ecdysteroids were detected in two out of 136 samples: from a Common Kestrel (*Falco tinnunculus*) that contained 58.9 nM 20-hydroxyecdysone (20E), and from a beech marten (*Martes foina*) that contained 40.9 nM dacryhainansterone (a phytoecdysteroid). Although these concentrations do not mean evidence for sustained biological or anabolic effects, 20E was reported to transactivate estrogen receptors β and α with EC_50_ values of 13.0 and 25.7 nM, respectively. Accordingly, our results show for the first time that ecdysteroids may be present in pharmacologically relevant concentrations in the blood of predominantly carnivorous species of birds and mammals. However, unlike in songbirds and bats which continuously feed on insects, detectable levels of ecdysteroids appear only rarely in the blood of opportunistic insectivores, therefore their health benefits remain questionable in artificially and occasionally insect-fed zoo animals.

## Introduction

Arthropod molting hormones, the so-called ecdysteroids are the only steroids produced by arthropods, with indispensable effects in their development, diapause, sexual maturation, reproduction and behavior [1,2]. Plants also biosynthesize such compounds, i.e., phytoecdysteroids, which may affect herbivorous insects [3]. In addition, ecdysteroids were experimentally shown to have anabolic effects in higher vertebrates, enhancing the metabolic utility of energy supplies, as well as promoting protein synthesis and tissue growth [4]. Importantly, wild-living populations of songbirds (Aves: Passeriformes) and bats (Mammalia: Chiroptera) were found to have low to high concentrations of these biologically active molecules in their blood [5,6]. It has also been shown most recently that grazing horses (Mammalia: Perissodactyla) acquire phytoecdysteroids which (or their derivatives) may appear in the circulation [7]. However, besides these high vertebrate orders, members of which constantly feed on either insects or plants, it is unknown if other vertebrates would also have food-derived, naturally acquired ecdysteroids in their circulation.

The aim of this work was to clarify if amniote vertebrates other than songbirds and bats may also have blood-borne ecdysteroids that were naturally or artificially acquired with their food. The target groups included reptiles, insectivorous mammals (e.g., hedgehogs), as well as small carnivores and primates that receive insect-containing diet in zoological gardens. In addition, anticoagulated blood was collected from wild-living opportunistic insect feeding mammals and non-passerine birds. These samples were screened for the presence of ecdysteroids with ultra-high performance liquid chromatography (UHPLC) and high-resolution quadrupole-orbitrap mass spectrometry.

## Materials and methods

### Origin of samples

Between 2022 and 2025, blood samples were taken from 136 amniote vertebrates. These included 30 regularly insect-fed zoo animals (27 mammals, 3 reptiles) kept in the Hannover Zoo (Germany), in the Budapest Zoo and in the Sóstó (Nyíregyháza) Zoo (Hungary). The insect diet contained yellow mealworm (*Tenebrio molitor*) larvae. In addition, 15 road-killed mammalian carnivores were also sampled *postmortem*. Their carcasses used in this study were routinely collected for research purposes, primarily by the ranger services of the Hungarian national park directorates. Based on necropsy condition, road-killed individuals were classified as fresh, that is, the time elapsed until discovery did not exceed 12–24 hours; all carcasses were frozen within approximately 1–2 hours after collection. Last but not least, 91 free-living wetland and predatory birds caught for ringing purposes were also sampled. Among these only juveniles (nestlings) were included except for gulls (Table 1). Blood was collected into EDTA-containing tubes by participating veterinarians during regular health care of zoo animals, or during handling with monitoring or ringing purposes in the case of wild-living birds. From living animals, approximately 0.1–0.5 mL of blood was collected from the caudal vein in reptiles and from the brachial/ulnar vein in birds. The samples were then immediately frozen and stored at −80 °C until analysis, thereby intending to minimize potential ecdysteroid degradation. Regarding mammals, blood samplings of hedgehogs, meerkats and skunks were performed during isoflurane anesthesia from the *vena cava cranialis*, and from foxes and drills using the saphenous vein (under ketamine*-*medetomidine anesthesia in the latter case). *Postmortem* blood samples were collected from the heart ventricle. Ethical permission for sampling wild-living birds was registered under No. GY/41/00001–10/2025 (issued by the Győr-Moson-Sopron County Government Office, Győr, Hungary). The post-mortem examination of the strictly protected Eurasian otter and wildcat was conducted under the PE-KTFO/508-4/2019 authority permit (HUN-REN Balaton Limnological Research Institute, Tihany, Hungary). Left-over blood samples collected during routine veterinary care of zoo animals were used for this study.

**Table 1:** Results of EDTA-anticoagulated blood collection: data of all samples screened for ecdysteroids with mass spectrometry. In each row, samples collected at the same location in the same month or on one occasion are shown. The age group of animals was “adult”, except when indicated as juvenile (juv) or nestling (pullus).

Vertebrate class	Order: species	Sex (age)	Country: location of sample collection	Year: month of sample collection	Total number of samples
**Mammalia**	Eulipotyphla: *Erinaceus roumanicus*	2M, 6F	Hungary: Budapest Zoo	2023: November	8
	Carnivora: *Vulpes zerda*	M	Hungary: Nyíregyháza Zoo	2024: September	1
	Carnivora: *Vulpes zerda*	2M, 2F	Hungary: Nyíregyháza Zoo	2025: March	4
	Carnivora: *Mephitis mephitis*	4M	Germany: Hannover Zoo	2024: March	4
	Carnivora: *Suricata suricatta*	8M	Germany: Hannover Zoo	2024: November	8
	Carnivora: *Felis silvestris*	M	Hungary: Csokonyavisonta	2025: March	1
	Carnivora: *Felis silvestris*	F	Hungary: Tószeg	2024: February	1
	Carnivora: *Felis silvestris*	M	Hungary: Drávaszentes	2025: January	1
	Carnivora: *Felis silvestris*	M	Hungary: Bódvaszilas	2025: March	1
	Carnivora: *Felis silvestris*	F (juv)	Hungary: Szín	2024: December	1
	Carnivora: *Felis silvestris*	F (juv)	Hungary: Jósvafő	2025: February	1
	Carnivora: *Martes foina*	F	Hungary: Kaposvár	2025: April	1
	Carnivora: *Martes foina*	M	Hungary: Zubogy	2022: February	1
	Carnivora: *Martes foina*	F	Hungary: Miskolc	2022: March	1
	Carnivora: *Martes foina*	F (juv)	Hungary: Aggtelek	2024: August	1
	Carnivora: *Lutra lutra*	M	Hungary: Siófok	2025: May	1
	Carnivora: *Lutra lutra*	F	Hungary: Isaszeg	2025: April	1
	Carnivora: *Lutra lutra*	F	Hungary: Nagykeresztúr	2025: January	1
	Carnivora: *Lutra lutra*	F (juv)	Hungary: Körpölye-Hugyag	2025: April	1
	Carnivora: *Lutra lutra*	F (juv)	Hungary: Sajószentpéter	2025: May	1
	Primates: *Mandrillus leucophaeus*	M	Germany: Hannover Zoo	2025: January	1
	Primates: *Mandrillus leucophaeus*	F	Germany: Hannover Zoo	2025: February	1
**Aves**	Anseriformes: *Anas platyrhynchos*	(5 pullus)	Hungary: Győr	2025: May	11
	Anseriformes: *Anser anser*	(2 pullus)	Hungary: Lipót	2025: June	2
	Charadriiformes: *Ch. ridibundus*	–	Hungary: Győr	2024: December	5
	Charadriiformes: *Ch. ridibundus*	–	Hungary: Győr	2025: February	5
	Charadriiformes: *Ch. ridibundus*	–	Hungary: Győr	2025: March	4
	Pelecaniformes: *Ardea cinerea*	(5 pullus)	Hungary: Dunaszeg	2025: May	5
	Suliformes: *Microcarbo pygmeus*	(5 pullus)	Hungary: Kapuvár	2025: May	5
	Suliformes: *Phalacrocorax carbo*	(7 pullus)	Hungary: Kapuvár	2025: May	7
	Accipitriformes: *Buteo buteo*	(1 pullus)	Hungary: Máriakálnok	2025: May	1
	Accipitriformes: *Buteo buteo*	(2 pullus)	Hungary: Rajka	2025: May	2
	Accipitriformes: *Buteo buteo*	(1 pullus)	Hungary: Hegyeshalom	2025: May	1
	Accipitriformes: *Buteo buteo*	(3 pullus)	Hungary: Töltéstava	2025: May	3
	Falconiformes: *Falco tinnunculus*	(10 pullus)	Hungary: Rajka	2025: June	10
	Falconiformes: *Falco tinnunculus*	(6 pullus)	Hungary: Kardoskút	2024: July	6
	Falconiformes: *Falco tinnunculus*	(4 pullus)	Hungary: Kardoskút	2024: August	4
	Falconiformes: *Falco vespertinus*	(20 pullus)	Hungary: Kardoskút	2024: July	20
**Reptilia**	Squamata: *Physignathus cocincinus*	M	Hungary: Nyíregyháza Zoo	2024: September	1
	Squamata: *Physignathus cocincinus*	M, F	Hungary: Nyíregyháza Zoo	2025: March	2

Abbreviations: M = male, F = female, juv = juvenile, Ch = *Chroicocephalus.*

### Sample preparation and calibration

All chemicals (water, methanol, acetonitrile, formic acid) for sample preparation and analysis with LC-MS grade were purchased from VWR (Radnor, PA, USA). Poststerone (20E)-oxime, used as an internal standard, was synthesized from poststerone as published earlier [8]. Blood samples were screened for the presence of ten natural ecdysteroids, as described previously [6]. The standards of these compounds were obtained from previous phytochemical studies [9,10]. For the investigated whole blood samples, 10 μL of Poststerone (20E)-oxime internal standard (IS) solution (1320 nM in methanol) was pipetted into a 1.7 mL centrifuge tube, followed by the addition of 50 μL of blood and 10 μL of methanol. For protein precipitation and enrichment of ecdysteroids, 980 μL of ice-cold methanol was added to the spiked blood samples. After vortexing, the sample was shaken for 10 min at room temperature and then taken to −20 °C for 20 min. Next, the sample was centrifuged at 4 °C for 15 min at 21,000 g (Universal 320 R, Hettich, Tuttlingen, Germany), and the upper phase was collected and dried under nitrogen at ambient temperature. The dried extract was dissolved in 70 μL of water/methanol (1/1, v/v) and centrifuged at 21,000 g at room temperature for 10 min, and the upper phase was collected for UHPLC-MS/HRMS analysis.

For matrix-matched external calibration, 10 μL of methanol (for blank) or a proper concentration of methanolic calibration mixture (for calibration points) was added to 50 μL of ecdysteroids-free blood samples, spiked with 10 μL of IS solution. The following steps were the same as those detailed above. After screening of 20-hydroxyecdysone (20E), polypodine B (pB), poststerone (pS), 14-deoxy-20-hydroxyecdysone (14d), ecdysone (E), ajugasterone C (AjC), calonysterone (Cal) and dacryhainansterone (Dac), only two samples were positive for 20E or Dac. For quantitative measurements, the concentration of the final calibration mixture was set to obtain 0, 1.04, 5.20, 10.40, 52.02, and 104.30 nM blood for 20E and 0, 1.08, 5.20, 10.81, 54.04, and 108.08 nM blood for Dac.

### Targeted ultra-high-performance liquid chromatographic-tandem high-resolution mass spectrometric (UHPLC-MS/HRMS) parameters

The targeted UHPLC-MS/HRMS measurement was performed by Waters Acquity I-Class UPLC (Milford, MA, UK) coupled to Thermo Scientific Orbitrap Exploris 240 (Waltham, MA, USA) mass spectrometer. MassLynx 4.1 (Milford, MA, USA) software was used for controlling the UHPLC and Xcalibur 4.5 software (Waltham, MA, USA) was used for MS/HRMS data acquisition. For quantitative evaluation of the UHPLC-MS/HRMS, raw files were processed using the integrated processing setup in Xcalibur 4.5.

The UHPLC was carried out on an Accucore C30 column (150 × 2.1 mm, 2.6 µm) with an equivalent guard column (10 × 2.1 mm, 2.6 µm; Thermo Fisher Scientific). The mobile phase A consisted of 0.1% formic acid aqueous solution, and mobile phase B was composed of acetonitrile with 0.1% v/v formic acid. The total run time was 15 min, and the following gradient program was used: 0 min 10% B held for 1 min; ramped to 40% B in 9 min; then ramped to 100% B in 0.5 min; held for 2.5 min; and, finally, returned to initial conditions within 0.5 min and equilibrated the column for 2.5 min. The flow rate was 0.4 ml/min during the analysis. The column temperature was maintained at 50 °C, and the injection volume was 7 µL. The autosampler was thermostated at 20 °C. All samples were measured four times.

The mass spectrometer was operated in positive mode using parallel reaction monitoring (PRM). The heated electrospray source was used with the following conditions: capillary temperature 300 °C, spray voltage 4.8 kV, RF lens 70%, sheath gas flow 45, spare gas flow 1, and auxiliary gas flow 5 in arbitrary units. In PRM mode with a resolution of 15,000 (FWHM), the AGC setting was defined as 1 × 10^5^ charges, and the maximum IT was set to 50 ms. The precursor ion window was set to 0.5 Da. In whole blood samples, the ecdysteroids were reliably confirmed by UHPLC-HRMS/MS analysis, which was based on literature data and the fragmentation pattern of the reference standards [5]. By means of PRM measurements on whole blood samples, 20E and Dac were unambiguously identified based on their retention times, exact masses of precursor and fragment ions, as well as fragmentation patterns. The mass tolerance for the processing of PRM spectra was set to 5 ppm. Five replicate quantitative analyses of samples were performed using external calibration with an internal standard, and the linear calibration curves for ecdysteroids were based on analyte/IS peak area ratios against concentrations. The LOQ values were determined to be 5 nM for the investigated compounds. The UHPLC-MS/HRMS measurements, containing the entire minimal dataset for this study, are available at this link: https://doi.org/10.17632/k9njjhjbcv.1.

## Results

Regularly insect-fed zoo animals successfully sampled for this study included Chinese water dragons (*Physignathus cocincinus*, n = 3), eastern hedgehogs (*Erinaceus roumanicus*, n = 8), desert (Fennec) foxes (*Vulpes zerda*, n = 5), striped skunks (*Mephitis mephitis*, n = 4), meerkats (*Suricata suricatta*, n = 8) and drills (*Mandrillus leucophaeus*, n = 2) (Table 1). These represented one reptilian and three mammalian orders (Squamata and Eulipotyphla, Carnivora, Primates, respectively). The analysis of blood samples from these zoo animals showed that they did not contain detectable levels of ecdysteroids.

**Fig 1:**
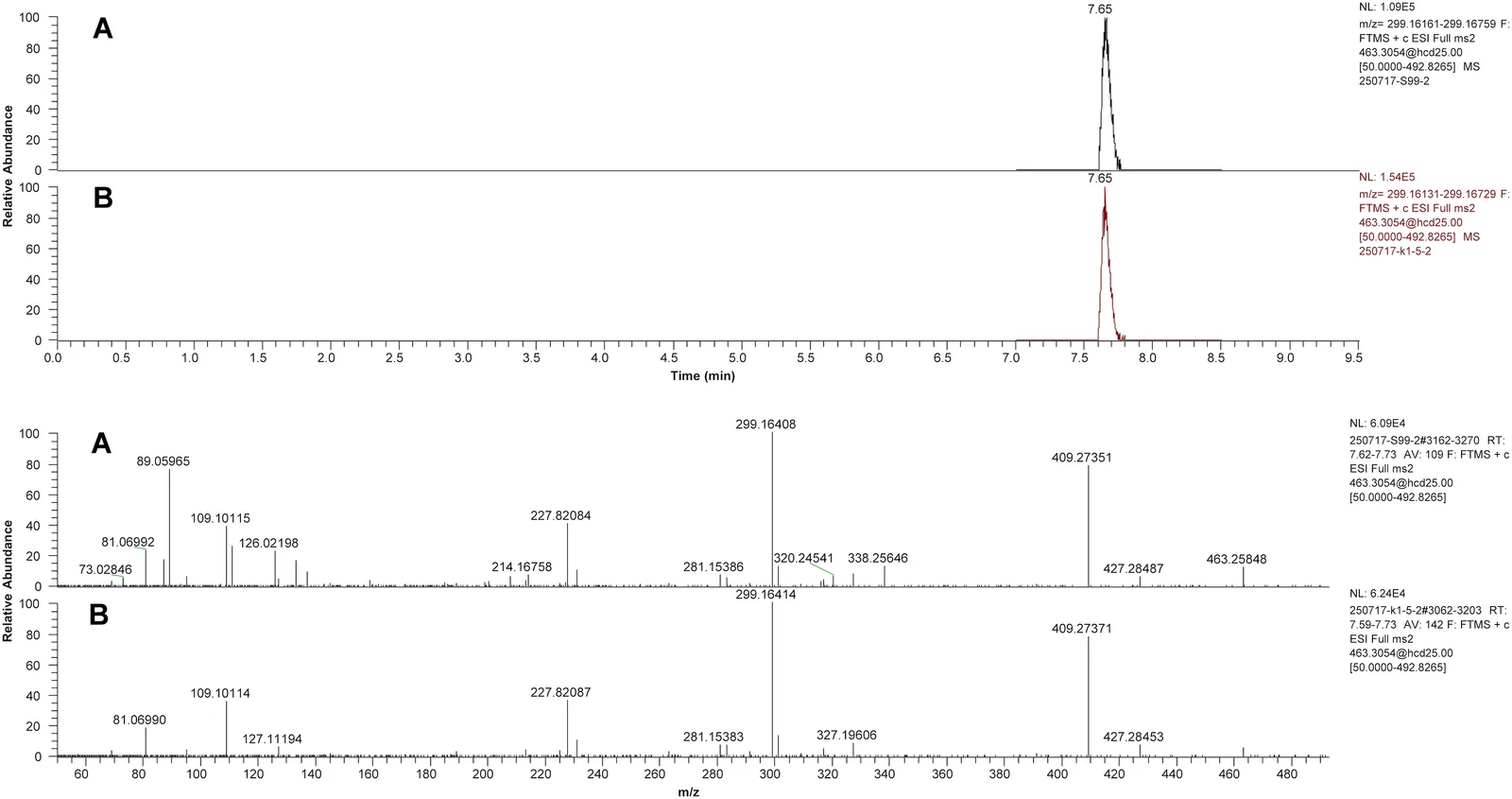
Upper panel: Extracted ion chromatogram of dacryhainansterone obtained by targeted UHPLC-MS/HRMS analysis of beech marten (A) and the fifth calibration sample (B). Lower panel: MS/HRMS spectra of Dac obtained by targeted UHPLC-MS/HRMS analysis of beech marten (A) and calibration sample (B). The Dac concentration obtained from the five replicate measurements was as follows in positive blood samples: 40.4, 40.2, 40.9, 41.6, and 41.4 nM.

Road-killed mammalian carnivores sampled *postmortem* in this study included six wild cats (*Felis silvestris*), four beech martens (*Martes foina*) and five Eurasian otters (*Lutra lutra*) (Table 1). Among these animals, 40.9 nM (CV: 1.4%) dacryhainansterone (a phytoecdysteroid) was detected in the blood of a beech marten (Fig 1) which was sampled in April, at Kaposvár, Hungary (Table 1).

**Fig 2:**
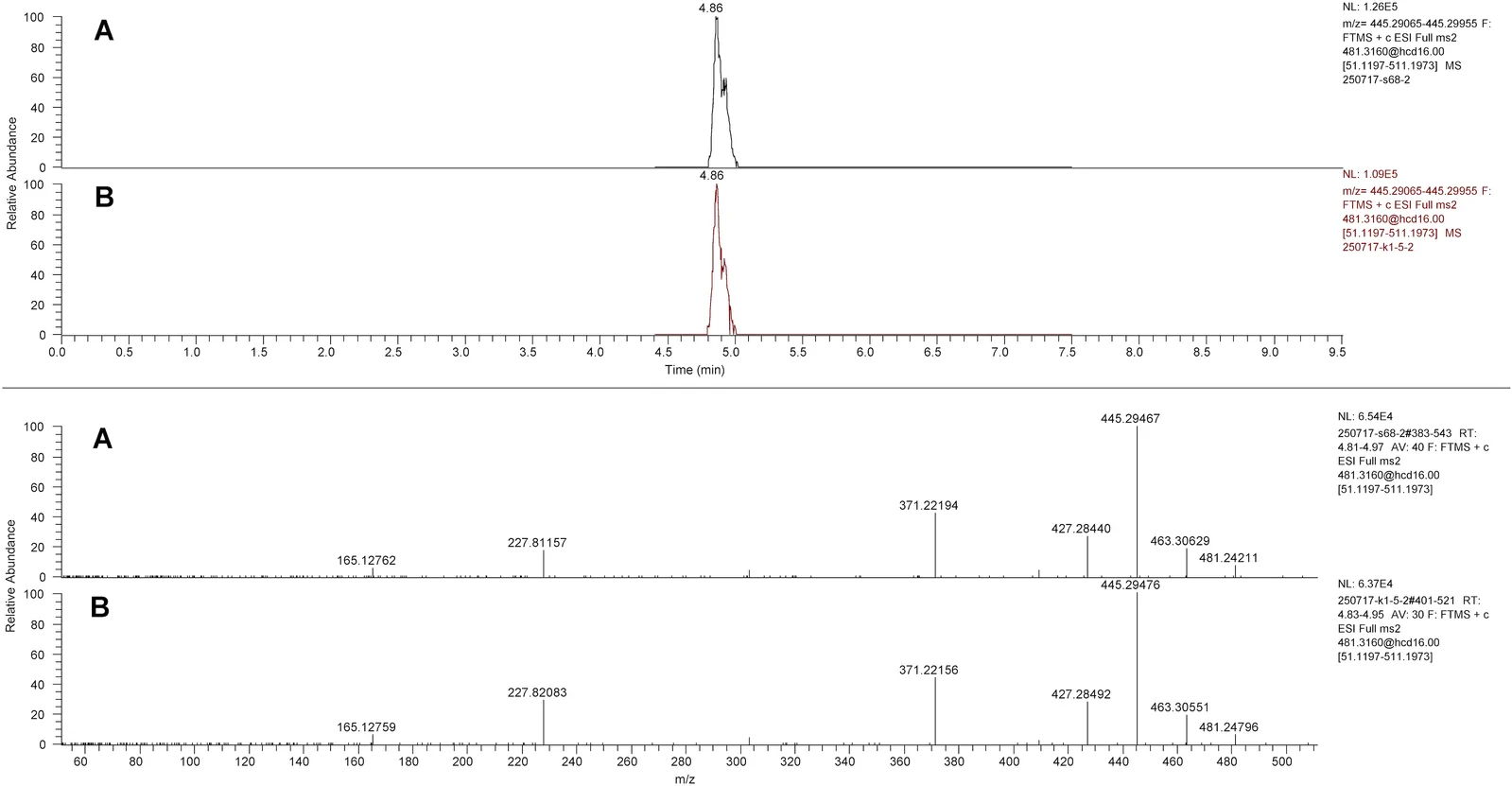
Upper panel: Extracted ion chromatogram of 20E obtained by targeted UHPLC-MS/HRMS analysis of Common Kestrel (A) and the fifth calibration sample (B). Lower panel: MS/HRMS spectra of 20E obtained by targeted UHPLC-MS/HRMS analysis of Common Kestrel (A) and calibration sample (B). The 20E concentrations obtained from the five replicate measurements in positive blood samples were as follows: 59.8, 58.0, 59.6, 58.5, and 58.8 nM.

Finally, blood samples were also drawn and analyzed from nine species of water-associated or predatory birds (n = 91) representing six avian orders (Table 1). In this group of samples, the blood of a Common Kestrel (*Falco tinnunculus*) contained 58.9 nM 20-hydroxyecdysone (CV: 1.2%) (Fig 2). This sample was collected in July, at Kardoskút, Hungary (Table 1).

Thus, the overall detection frequency was 1.47% (2 positives out of 136 samples; 95% confidence interval: 0.18–5.21%).

## Discussion

Previously, the presence of food-derived ecdysteroids was demonstrated in blood samples of the great majority of examined passerine birds and bats which continuously feed on insects [5,6]. While these biologically active compounds may have anabolic (energizing and muscle growth-promoting) effects that are particularly beneficial considering the high energy need and muscle performance during active flying, this phenomenon also turned out to be advantageous in potentially shortening the duration of blood-feeding among bird ectoparasites such as ticks [11].

In some of the songbirds examined previously, the concentration of blood-borne ecdysteroids reached very high levels, occasionally exceeding 2000 nM concentrations of 20-hydroxyecdysone, but in other individuals this was significantly lower, below 20 nM [5]. These values indicate a higher concentration range in songbirds than the level detected in the kestrel nestling of this study (58.9 nM). On the other hand, the levels of ecdysteroids were lower in bats than in songbirds. For instance, while in songbirds dacryhainansterone could reach as high as 7500 nM, in bats its levels were orders of magnitude lower and the mean dacryhainansterone concentration was only 25.2 nM [6]. The latter is similar to the level of the same plant-derived (phyto-) ecdysteroid found in this study in the beech marten (40.9 nM) which was the only mammal that showed a detectable concentration of any ecdysteroid in its blood. The lowest limit of detection (LOD) was obtained for 20-hydroxyecdysone (20E), at 0.5 nM, whereas the detection limit for the other compounds was 1 nM. This means that our negative results do not exclude the presence of concentrations below these values. Based on currently available literature data, it is unclear if such low blood concentrations could hold any relevance.

Considering the molting hormones biologically most active in insects, i.e., the “cardinal ecdysteroids” (including both 20-hydroxyecdysone and dacryhainansterone), these were detected over the whole evaluation period (March to October) in most songbird species [6]. In this study, the blood from the kestrel nestling contained 20-hydroxyecdysone in the summer, in line with the high ratio of insects in their diet during that period of the year [12]. On the other hand, the beech marten in the blood of which dacryhainansterone was detected, was sampled during the spring. This finding is probably the consequence of what was observed among beech martens in the study region, i.e., that approx. 70% of the consumed biomass is plant material during the springtime [13]. In addition, this phytoecdysteroid may have also been ingested by the beech marten via the food chain, i.e., with plant-feeding insects in its diet. In support of this notion, invertebrates including insects are present in the highest ratio in the food of beech martens during springtime [13]. Further, while the postmortem detection of this compound in the beech marten raises interesting questions concerning postmortem metabolism or redistribution, nothing is currently known on the pharmacokinetic behavior of this compound, therefore only further speculations could be made in this regard. It may also worth mentioning that little is currently known on the stability of dacryhainansterone during sample processing and storage. This warrants further related studies to evaluate the possibility of its transformation into other ecdysteroids, not measured in our work, and/or a possible metabolic or chemical formation of this compound from another, more abundant ecdysteroid.

Based on the sample selection criteria applied in this study, it is not a question that at the time of blood sampling, or the day before, ecdysteroids were present in the food of all zoo animal individuals analyzed here. Therefore, we hypothesize that the most important factors influencing their detectability in the blood samples obtained here were their (1) quantity, (2) absorption, and (3) metabolism or clearance from the gut/circulation. (1) In contrast to songbirds and bats, which constantly feed on insects and similar invertebrates, among opportunistically insectivorous vertebrates analysed in this study, the quantity of food-derived ecdysteroids in the gut must have been lower. Concerning (2) their absorption from the gut into the circulation, it was shown that small birds and bats have significantly shorter small intestines and less absorptive surface area than similarly sized nonflying mammals. To compensate for this, in small birds and bats, an enhanced paracellular pathway exists for intestinal absorption of water-soluble nutrients [14]. The many hydroxyl groups of ecdysteroids confer them moderate to even high water solubility, unlike the case of typical steroid lipids [15,16]. Therefore, in small songbirds and bats as flying vertebrates, this mechanism of paracellular absorption could probably contribute to the effective intestinal absorption of ecdysteroids [6], unlike in non-flying vertebrates and larger bird species studied here. (3) Concerning the metabolism and elimination of ecdysteroids, related studies were focusing on 20E, and it was found that both in rodents and humans the elimination is fast, and the overall bioavailability after oral administration is very low, just a few percents [17]. Major metabolic pathways involve a sidechain cleavage between C20,22, leading to the formation of poststerone derivatives, and a reduction of C14 and/or the enone B-ring [17]. We may hypothesize that compounds with a more extended conjugation, such as dacryhainansterone, are more resistant to this latter metabolic step. This would offer a possible explanation why this not so widespread compound was among the most abundant ones in passerine birds [5], and why it was the only ecdysteroid other than 20E that could be identified among the samples tested here. Despite the very low hit-rate (2 positive samples out of 136) and relatively low ecdysteroid concentrations (40.9 and 58.9 nM) observed, it is worth mentioning that such concentrations are well within the pharmacologically relevant range in vertebrates. For example, the anabolic effect of 20E in rats was linked with its action on estrogen receptor beta (ERβ), while it was less potent but also active on estrogen receptor alpha (ERα): in an *in vitro* transactivation assay, EC_50_ values of 13.0 and 25.7 nM were measured for Erβ and ERα, respectively. On the other hand, it must still be noted that while the detected concentrations fall within ranges reported to activate estrogen receptors in vitro, the sporadic nature of detection strongly suggests that sustained biological effects in opportunistic insectivores are unlikely [18].

Considering the worsening resource shortage of human food supplies (e.g., due to climate change affecting livestock-derived food productivity, and growing demands owing to overpopulation), several foods have been proposed as alternatives, with insects receiving the most attention [19]. Besides having high protein contents [20], insects surpass conventional food groups by excellent production efficiency [21]. In light of these developments and future trends, it is not surprising that the nutritional values of insects represent a hot topic in science. However, despite their presence, ecdysteroids are usually omitted from studied components of insect food [22]. Here, no detectable ecdysteroids were shown to be present in the blood of two primates (drills) regularly receiving insect food. This, to our knowledge, is the first assessment of these substances in any primates, with data highly relevant to the consumption of insect larvae by humans. These preliminary findings indicate that, under typical dietary conditions, insect-derived ecdysteroids are unlikely to accumulate in the bloodstream of primates. Nevertheless, controlled dietary intervention studies are required to rigorously evaluate this hypothesis. In addition, comprehensive analysis of urine and fecal samples is essential to better characterize the absorption, metabolism, and excretion of ecdysteroids and to obtain more reliable conclusions.

Unlike in the case of insect-fed zoo animals, dietary exposure to insect-derived ecdysteroids immediately before sampling cannot be confirmed for all opportunistically insectivorous wild animals studied here. This should be taken into account as a limitation when interpreting the negative findings. Last but not least, because the blood samples analyzed in this study originated from taxonomically diverse vertebrates, potential differences in matrix effects among animal groups cannot be excluded. Owing to the limited sample volumes available, a separate evaluation of matrix effects for different taxonomic groups was not feasible. Future studies with sufficient sample volumes should therefore address this aspect to further refine the analytical methodology.

In summary, it was demonstrated for the first time that ecdysteroids may also be present in the blood of predominantly carnivorous species of birds and mammals. However, unlike in passerine birds and bats which continuously feed on insect, detectable levels of these anabolic substances appear only rarely in the blood of opportunistic insectivores. This is probably related to the quick clearance of ecdysteroids in vertebrates. The pharmacologically relevant concentrations observed in the two positive samples, however, leave open the question about the effects ecdysteroid exposure might have in occasionally insect-fed zoo animals.
